# Measurement and interpretation of electrocardiographic QT intervals in murine hearts

**DOI:** 10.1152/ajpheart.00459.2013

**Published:** 2014-04-04

**Authors:** Yanmin Zhang, JingJing Wu, James H. King, Christopher L.-H. Huang, James A. Fraser

**Affiliations:** ^1^Physiological Laboratory, University of Cambridge, Cambridge, United Kingdom;; ^2^Heart Centre, Northwest Women's and Children's Hospital (formerly the Shaanxi Provincial Maternity and Children Healthcare Hospital), Xi'an, China; and; ^3^Centre for Ion Channel Research and Department of Cardiovascular Diseases, Union Hospital, Huazhong University of Sciences and Technology, Wuhan, China

**Keywords:** action-potential duration, ECG, QT interval, mouse heart

## Abstract

Alterations in ECG QT intervals correlate with the risk of potentially fatal arrhythmias, for which transgenic murine hearts are becoming increasingly useful experimental models. However, QT intervals are poorly defined in murine ECGs. As a consequence, several different techniques have been used to measure murine QT intervals. The present work develops a consistent measure of the murine QT interval that correlates with changes in the duration of ventricular myocyte action potentials (APs). Volume-conducted ECGs were compared with simultaneously recorded APs, obtained using floating intracellular microelectrodes in Langendorff-perfused mouse hearts. QT intervals were measured from the onset of the QRS complex. The interval, Q-APR_90_, measured to the time at 90% AP recovery, was compared with two measures of the QT interval. QT1 was measured to the recovery of the ECG trace to the isoelectric baseline for entirely positive T-waves or to the trough of any negative T-wave undershoot. QT2—used extensively in previous studies—was measured to the return of any ECG trough to the isoelectric baseline. QT1, but not QT2, closely correlated with changes in Q-APR_90_. These findings were confirmed over a range of pacing rates, in low K^+^ concentration solutions, and in *Scn5a*+/Δ*KPQ* hearts used to model human long QT syndrome. Application of this method in whole anesthetized mice similarly demonstrated a prolonged corrected QT (QTc) in *Scn5a*+/Δ*KPQ* hearts. We therefore describe a robust method for the determination of QT and QTc intervals that correlate with the duration of ventricular myocyte APs in murine hearts.

the duration of ventricular depolarization and repolarization is reflected in the ECG QT interval (1, 13). It is important for the diagnosis of inherited and iatrogenic QT disorders associated with increased risks of polymorphic ventricular tachycardia or Torsades de Pointes that may present as long or short QT syndromes. Transgenic mouse hearts have proven increasingly useful in modeling human cardiac arrhythmic disease. However, mice differ from humans in aspects of cardiac electrophysiology that have implications for ECG interpretation. Murine hearts show shorter electrocardiographic RR intervals, reflecting higher heart rates (600 beats/min).

Murine QRS-T complexes reflect considerably shorter action-potential (AP) waveforms lacking plateau phases, resulting in indistinct ST segments and T-waves (12). Nevertheless, the T-wave is often assumed to end at its return to the isoelectric baseline (5, 9). However, where the T-wave in some mouse ECG traces shows an apparent negative undershoot, in addition to its positive component, this approach would substantially increase estimates of the QT interval or preclude determination of QT intervals at high heart rates when the P-wave often becomes superimposed upon the terminal phase of the previous negative undershoot (12).

Previous murine studies had adopted different empirical QT estimation methods, including determining: *1*) the time taken to reach 95% recovery for the ECG deflection to return to baseline (10); *2*) the time to the end of the T-wave, itself defined as the point at which the T-wave time course deviates from the tangent to the steepest slope of the time course of T-wave recovery (9); *3*) the time at which the QT segment returns to baseline (3, 6, 9a); and *4*) the point of convergence of T-waves and their first derivative on a signal-averaged ECG recording (14). These various approaches met with variable success, leading to doubts as to whether ECG analysis of this kind could quantify murine AP parameters (4).

The present paper develops a simple empirical approach for measuring the QT interval in mouse ECGs by comparing ECGs with simultaneous intracellular recordings of AP waveforms in Langendorff-perfused hearts. It then tests the resulting criteria over a range of pacing rates, and under conditions of altered QT interval in low K^+^ concentration ([K^+^]) solutions and in *Scn5a*+/Δ*KPQ* hearts modeling long QT syndrome. It then further compares in vitro recordings with QT intervals recorded in intact, anesthetized *Scn5a*+/Δ*KPQ* mice.

## METHODS

### 

#### Experimental animals.

Experiments were conducted using wild-type (WT) and *Scn5a*+/Δ*KPQ* mice, inbred on a 129/Sv genetic background, aged 3–6 mo, housed in cages at 21 ± 1°C with 12-h light/dark cycles. All procedures were performed in institutional premises, approved under the UK Animals (Scientific Procedures) Act (1986), under UK Home Office project licence PPL number 80/1974, approved by a university Ethics Review Board. Accordingly, procedures were also in conformity with the Guide for the Care and Use of Laboratory Animals, published by the U.S. National Institutes of Health (NIH publication number 85-23, revised 1996).

#### Simultaneous epicardial ventricular AP and volume-conducted electrocardiographic recordings from intact Langendorff-perfused hearts.

Mice were killed by cervical dislocation [Schedule 1: UK Animals (Scientific Procedures) Act 1986]. Their aortas were cannulated, and the heart was perfused at a constant flow rate of 3 ml/min (Bredel peristaltic pumps, model 505S; Watson-Marlow, Falmouth, Cornwall, UK) with Krebs-Henseleit (KH) solution (in mM: NaCl 119, NaHCO_3_ 25, KCl 4, KH_2_PO_4_ 1.2, MgCl_2_ 1, CaCl_2_ 1.8, glucose 10, Na-pyruvate 2, pH adjusted to 7.4), bubbled with 95% O_2_/5% CO_2_ (British Oxygen, Manchester, UK) on a Langendorff system. The KH solution was passed through a 5-μm filter (Millipore, Watford, UK) and warmed to 37°C using a water jacket and circulator (model C-85A; Techne, Cambridge, UK). Hearts were laid down with their anterior surfaces facing upward in a homemade, warmed bath chamber. Only hearts that regained their pink color and showed 1:1 atrioventricular conduction with intrinsic activity and after 10–15 min perfusion for stabilization were then subjected to further electrophysiological testing.

A floating microelectrode holder was constructed from a thin, coiled silver wire (0.4 mm in diameter) and connected to a 2-mm connecter. A glass micropipette was drawn from borosilicate glass to a very fine tip and filled immediately before use with 3 M KCl. The pipette was cut above its shoulders, and the remaining shank was discarded. The microelectrode resistances were 15–25 MΩ. The chlorided end of the silver wire was inserted into the micropipette, with which impalements were made close to the midpoint between ventricular apex and base, and connected to a high-input impedance direct-current microelectrode amplifier system (University of Cambridge, Cambridge, UK). The signals were displayed, digitized, and analyzed using Spike2 (Cambridge Electronic Design, Cambridge, UK). Conversion of the analog input to digital signals was performed using a model Micro1401 interface (Cambridge Electronic Design) connected to an IBM-compatible computer. Spike2 software (Cambridge Electronic Design) was used to record and subsequently analyze ECG recordings. The entire apparatus was mounted on a vibration-isolation platform in a grounded Faraday cage. APs showing straight upstrokes, with AP amplitudes >75 mV, maximum rates of rise >85 mV/ms, and resting potentials between −80 and −65 mV, were used for further analysis.

Volume-conducted ECGs were recorded simultaneously with the AP recordings. Three-needle electrodes were immersed in the superfused bath flanking the isolated heart. Signals were amplified and filtered by a model NL104A amplifier (NeuroLog; Digitimer, Hertfordshire, UK) and a model NL125/126 filter (set to a bandwidth of 10–5,000 Hz). Conversions of analog input to digital form used a model 1401+ interface (Cambridge Electronic Design) connected to an IBM-compatible computer. Spike2 software (Cambridge Electronic Design) was used to record and subsequently analyze ECG recordings.

An initial series of experiments studied hearts in sinus rhythm. Further experiments assessed the ECG measured under conditions of regular stimulation at cycle lengths (CL) of 200, 167, and 143 ms, at which 50 APs were recorded for each CL. This used a bipolar, platinum-stimulating electrode (1 mm interpole spacing) placed against the right atrial epicardium, delivering square-wave stimuli (Grass S48 stimulator; Grass-Telefactor, Slough, UK) of 2-ms duration and amplitudes of twice diastolic excitation threshold. The experiments also examined the effect of reducing the extracellular [K^+^] from normokalemic (4 mM) to hypokalemic (3 mM) levels in the KH perfusate to investigate the effects of conditions that would prolong the QT interval and its measurement.

#### Measurements from the ECG and intracellular AP traces.

The duration of ventricular electrophysiological activity was determined by three measurements from the ECG and the AP traces. In each case, peaks and recovery times were obtained from direct readouts from successive averages of five data points, obtained by software cursors successively moved along the time axis. In the ECG traces, the position of the isoelectric baseline was defined as falling between the end of the upright P-wave and the beginning of the QRS in the volume-conducted ECGs, as on previous occasions (15). The beginning of ventricular electrical activity was defined by the onset of the QRS in the ECG trace, where it deviated from this isoelectric baseline. The QT interval was then calculated by two methods. *1*) QT1 was measured to the time at which the ECG trace first reached its minimum value; this was the isoelectric baseline when the T-wave only showed positive components or the point of maximum undershoot where records showed a negative undershoot. *2*) QT2 was defined as the time at which the T-wave reached the isoelectric baseline. *3*) For comparison with ECG records, the Q-APR_90_ was defined as the time intervening between the start of the ECG QRS complex and the time of the APR_90_, the point of 90% recovery of the ventricular AP from its peak to the isoelectric baseline in the intracellular microelectrode recordings. Corrected QT (QTc) intervals were then obtained using the formula QTc = QT/(RR/100)^1/2^ to give QTc1, QTc2, and Q-APR_90_c respectively (11).

#### In vivo ECG recordings from mice under terminal anesthesia.

Independent, in vivo ECG studies were performed on mice anesthetized with a dose of 0.10 ml/10 g body wt of either: *1*) a solution comprising 1.8 ml of 100 mg/ml ketamine hydrochloride (Ketaset; Fort Dodge Animal Health, Southampton, UK), 0.35 ml of 23.32 mg/ml xylazine hydrochloride (Rompun; Bayer, Leverkusen, Germany), and 2.85 ml of sterile phosphate-base solution or *2*) 24 mg/ml ip Avertin (2,2,2-tribromoethanol; Sigma, Poole, Dorset, UK) into the left peritoneal cavity, respectively, 15 and 5 min before recording. The anesthetized mice were placed on a heating pad with continuous monitoring of body temperature for three-lead ECG measurements in lead II for over 10 min using subcutaneous needle electrodes and a PowerLab 26T system (ADInstruments, Oxfordshire, UK). Recordings (16 bit, 2 kHz/channel) were analyzed using the Chart v6.0 program (ADInstruments). Recordings were filtered between 0.5 and 500 Hz.

#### Data analysis and statistics.

Data are expressed as means ± SE. The numbers (*n*) denote either numbers of whole hearts or the number of cells. The sets of data were compared using ANOVA with post hoc Tukey's honestly significant difference test, as well as regression analysis (SPSS software; SPSS, Chicago, IL). *P* < 0.05 was considered statistically significant.

## RESULTS AND DISCUSSION

**Fig. 1.**
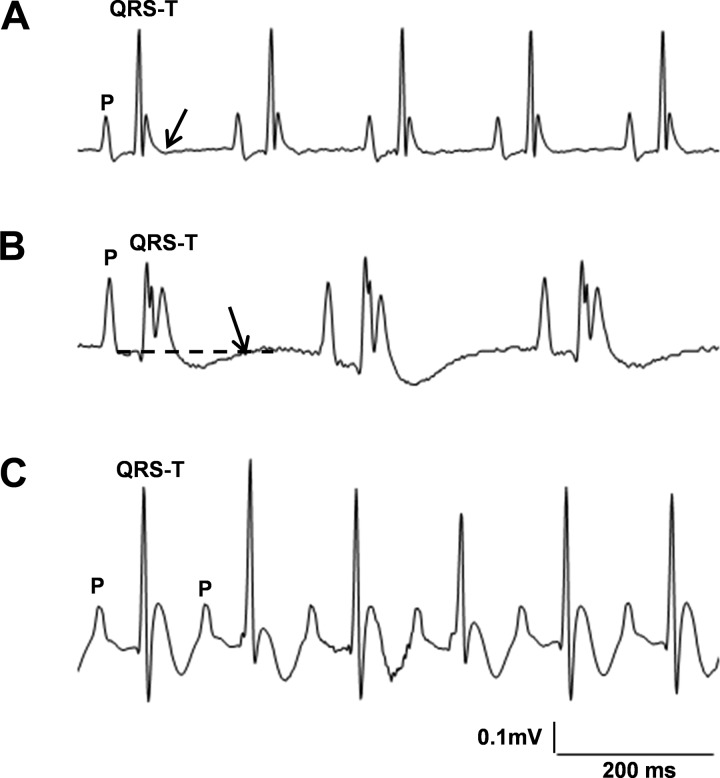
ECG patterns from anesthetized mice show marked variability. *A*: recorded from a wild-type (WT) mouse; the QRS-T complex ends with an upright T-wave that then returns to the isoelectric baseline (arrow). *B*: recorded from an *Scn5a+/ΔKPQ* mouse; the positive part of the T-wave is followed by a negative undershoot before returning (arrow) to the isoelectric baseline (dashed line). *C*: recorded in a WT mouse, 10 min after administration of isoprenaline (2.0 mg/kg ip); the T-waves show negative undershoot components, not fully separable from the succeeding P-waves.

Figure 1 displays typical ECGs from a range of Avertin-anesthetized mice studied under similar conditions. Isoelectric baselines were determined between the end of the P-wave and the beginning of the QRS (16). The beginning of ventricular electrical activity was defined by the beginning of the QRS complex. As reported previously in both mouse and rat, the beginnings of the T-waves were often indistinct, likely reflecting short or absent AP plateau phases (12, 17).

Figure 1*A* shows an upright ECG T-wave with a clear-cut decay to isoelectric baseline. In contrast, in Fig. 1*B*, the positive component of the T-wave was followed by a prolonged, negative undershoot. Inclusion of the latter waveform would thus substantially increase estimates of the QT interval (40 ms vs. 130 ms, respectively). Figure 1*C* shows T-waves with negative undershoot components, not clearly separable from the succeeding P-waves, which would complicate determination of the end of the T-wave.

**Fig. 2.**
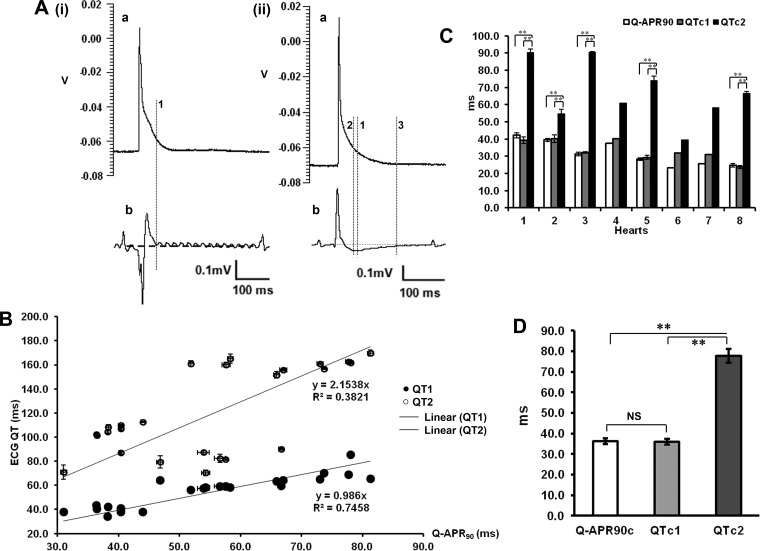
Relationships between intracellular action-potential (AP) duration and volume ECG QT intervals in WT hearts. *A*, (*i*) and (*ii*): APs (*a*) and the corresponding ECG recordings (*b*). *Cursor 1* indicates the AP at 90% recovery (Q-APR_90_). *Cursor 2* is placed at the minimum value of a late negative undershoot (QT1). *Cursor 3* indicates the point where the undershoot component regains the isoelectric baseline (QT2). V, volts. *B*: plots of QT1 and QT2 intervals against Q-APR_90_ values observed and their respective regression lines. *C*: summarization of corrected Q-APR_90_ (Q-APR_90_c) and corrected QT1 and QT2 (QTc1 and QTc2, respectively), resulting from 8 individual hearts. Results from *hearts 1–8* were obtained in the course of recording from *n* = 6, 4, 3, 1, 5, 1, 1, and 3 cells, respectively, of which, ECG records in *hearts 2* and *6* showed a kinetic pattern, generally similar to that in *A*, (*i*), *b*, whereas the remainder showed more complex kinetic patterns similar to that in *A*, (*ii*), *b*. *D*: summarization of mean (±SE) Q-APR_90_c, QTc1, and QTc2 values. **P < 0.01.

Figure 2*A* compares typical intracellular ventricular APs (*a*), obtained in WT hearts, with simultaneously recorded, volume-conducted ECGs (*b*). The latter showed positive-going T-waves, followed by direct decays [Fig. 2*A*, (*i*)] or by prolonged, negative undershoots [Fig. 2*A*, (*ii*)]. Different definitions of the QT interval, as depicted by the cursors, were compared with predictions from AP recordings. Thus *cursor 1* shows Q-APR_90_ in the AP trace. *Cursor 2* depicts QT1, extending to the point at which the ECG trace regained the isoelectric baseline in records where the T-wave only showed positive components or where any prolonged, negative undershoots reached their minima. In this example, QT1 was in agreement with Q-APR_90_ in each case [within ∼9.8 ms in Fig. 2*A*, (*ii*)]. In contrast, QT2, which extended to full recovery to the isoelectric baseline (*cursor 3*), gave marked 104.7-ms differences from Q-APR_90_ with the waveform depicted in Fig. 2*A*, (*ii*).

Figure 2*B* plots QT1 and QT2 against Q-APR_90_ in each of 24 myocytes from eight WT hearts, including means and SE of the mean where the latter exceeded the sizes of the data points themselves. Each point shows ECG and intracellular AP parameters obtained simultaneously and averaged over the full duration of each successful cell impalement. The regression lines showed that QT1 closely correlated with Q-APR_90_ [QT1 = 0.986 × Q-APR_90_; regression coefficient (*r*) = 0.863]. In contrast, QT2 values correlated poorly with Q-APR_90_ (QT2 = 2.15 × Q-APR_90_; *r* = 0.618), as demonstrated previously (4).

Figure 2*C* illustrates the closeness or otherwise of Q-APR_90_c, QTc1, and QTc2 for each of the eight hearts that were studied. In each of these individual hearts, Q-APR_90_c and QTc1 values were statistically indistinguishable (*P* > 0.05, *n* = 8). In contrast, QTc2 values were consistently larger than both Q-APR_90_c and QTc1 (Fig. 2*C*). Concordant results emerged from comparisons between Q-APR_90_c and QTc1 (*P* > 0.05) but not between these measures and QTc2 (*P* < 0.01 in both cases) over the entire set of hearts (Fig. 2*D*). Hence, QTc1 correlated well with Q-APR_90_c over a wide range of values, whereas QTc2 is larger than Q-APR_90_c and correlates with it poorly, especially when the T-wave has negative components.

Measurements of Q-APR_90_, QT1, and QT2 intervals, averaged over 50 beats, were then repeated during pacing at CL of 200, 167, and 143 ms. Means and SE at the three pacing rates were similar for Q-APR_90_ (41.04 ± 0.09, 41.55 ± 0.05, and 41.02 ± 0.04 ms, respectively) and QT1 (42.78 ± 0.17, 42.47 ± 0.19, and 41.66 ± 0.46 ms). In contrast, both means and the SE of QT2 varied markedly at the three pacing rates (109.56 ± 0.74, 101.76 ± 0.77, and 73.03 ± 0.58 ms, respectively) and differed significantly from Q-APR_90_ in each case.

Further measurements of Q-APR_90_, QT1, and QT2 were made before and after acute alterations in AP durations, produced by replacing normokalemic with hypokalemic perfusate, previously shown to produce prolongation of AP duration as recorded using monophasic AP electrodes (8). Hypokalemia increased Q-APR_90_ from 40.89 ± 1.08 (four cells in one heart) to 44.27 ± 0.72 ms, respectively (eight cells in one heart). Simultaneously recorded QT1 values similarly increased, from 41.02 ± 2.49 to 53.08 ± 0.37 ms. In contrast, the corresponding QT2 values reduced from 95.79 ± 3.22 to 89.00 ± 3.89 ms, respectively.

**Fig. 3.**
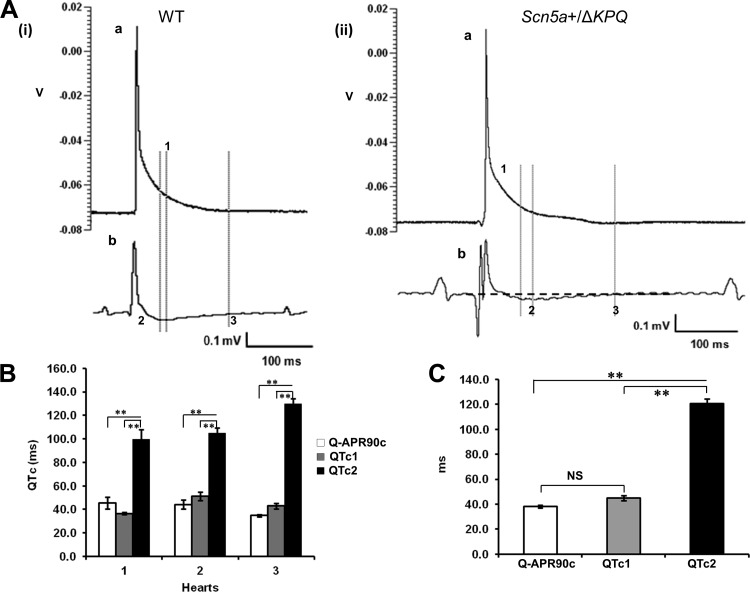
Relationships between intracellular AP duration and volume ECG QT intervals in *Scn5a*+/Δ*KPQ* hearts. *A*: comparison of simultaneous recordings of a typical AP from a single ventricular myocyte (*a*) with the corresponding ECG trace (*b*) from WT (*i*) and *Scn5a*+/Δ*KPQ* (*ii*). *B*: comparison of Q-APR_90_c, QTc1, and QTc2 in individual *Scn5a*+/Δ*KPQ* hearts. *C*: summarization of mean (±SE) Q-APR_90_c, QTc1, and QTc2 values. **P < 0.01.

QT intervals were then quantified in murine *Scn5a*+/Δ*KPQ* hearts containing the *ΔKPQ* (1,505–1,507) gain-of-function *Scn5a* deletion, modeling human long QT syndrome and expected to demonstrate chronic QT-interval prolongation (7, 18). Ventricular APs were obtained from 40 cells in three *Scn5a*+/Δ*KPQ* hearts. An example is shown in Fig. 3*A*, (*ii*), for comparison with a WT recording [Fig. 3*A*, (*i*)]. These showed greater AP durations than WT (Q-APR_90_c values of 41.47 ± 3.37 in *Scn5a*+/*ΔKPQ* vs. 36.33 ± 1.45 in WT, *P* < 0.05). Simultaneously recorded, volume-conducted ECGs similarly demonstrated increased values of QTc1 (43.58 ± 4.22) and QTc2 (111.3 ± 5.91) relative to WT [35.99 ± 1.33 (*P* < 0.05) and 66.92 ± 6.21 (*P* < 0.01), respectively]. As for the WT hearts (Fig. 2), mean QTc1 values in *Scn5a*+/Δ*KPQ* hearts (Fig. 3*C*) were not significantly different from Q-APR_90_c (43.58 ± 4.22 vs. 41.47 ± 3.37, *P* > 0.05), whereas their mean QTc2 value (111.3 ± 5.91) was significantly larger than both Q-APR_90_c and QTc1 (*P* < 0.01 in each case).

Finally (Table 1), ECGs were recorded from intact, anesthetized—as opposed to isolated, perfused—*Scn5a*+/Δ*KPQ* and WT mice. These were statistically indistinguishable (*P* > 0.05) within groups under either Avertin (*n* = 19 and 26, respectively) or ketamine anesthesia (*n* = 10 and 23). However, *Scn5a*+/Δ*KPQ* showed consistently greater QTc1 than WT (44.56 ± 2.23 and 45.42 ± 3.00 in *Scn5a*+/Δ*KPQ* vs. 33.60 ± 1.87 and 30.75 ± 1.50 ms in WT, *P* < 0.05 in both cases), in agreement with findings in isolated, perfused hearts.

**Table 1.** Summary comparing QTc intervals in anesthetized mice during ECG recordings

Condition	Genotype	*n* = Hearts	QTc, ms
Avertin	WT	26	33.60 ± 1.87*
	*Scn5a*+/Δ*KPQ*	19	44.56 ± 2.23*
Ketamine	WT	23	30.75 ± 1.50^†^
	*Scn5a*+/Δ*KPQ*	10	45.42 ± 3.00^†^

Note that similar symbols differ from each other at *P* < 0.05. QTc, corrected QT; WT, wild-type.

Thus the time from the start of ventricular activity to the time of minimum voltage (QT1) is a stable and repeatable measurement of the murine QT interval that correlates with the duration of intracellularly recorded APs.

## GRANTS

Support for this work was provided by a Biotechnology and Biological Sciences Research Council David Phillips Fellowship, held by J. A. Fraser (BB/FO23863/1 to J. A. Fraser and Y. Zhang); the Natural Science Foundation of China (30371571, 30830051, and 30672209 to J. Wu and Y. Zhang); the Medical Research Council; the Wellcome Trust (to C. L-H. Huang); and the British Heart Foundation (to C. L-H. Huang and J. H. King).

## DISCLOSURES

The authors have no conflicts of interest to disclose.

## AUTHOR CONTRIBUTIONS

Author contributions: Y.Z. and J.A.F. conception and design of research; Y.Z., J.W., and J.H.K. performed experiments; Y.Z., J.W., and J.H.K. analyzed data; Y.Z. and J.A.F. interpreted results of experiments; Y.Z. and J.A.F. prepared figures; Y.Z. and J.A.F. drafted manuscript; Y.Z., J.W., J.H.K., C.L-H.H. and J.A.F. edited and revised manuscript; C.L-H.H., and J.A.F. approved final version of manuscript.
